# Supplementary material for: Small Molecule Targeting Immune Cells: A Novel Approach for Cancer Treatment
Source: Biomedicines. 2023 Sep 24;11(10):2621. doi: 10.3390/biomedicines11102621 (PMC10604364; doi:10.3390/biomedicines11102621)
Supplement: Supplementary file 1 [file biomedicines-11-02621-s001.zip › biomedicines-2501780-supplementary.pdf]

**Supplementary Table S1. Clinical studies of combination therapy for cancer treatment**

| Combination studies | Phase | Cancer type | Registration number | Status | Inventors |
|---|---|---|---|---|---|
| DNX2401<br>Temozolomide | I | Glioblastoma multiforme<br>Recurrent tumor | NCT01956734 | Completed | Clinica universidad de Navarra |
| CVA21<br>Ipilimumab | I | Uveal melanoma<br>Liver metastases | NCT03408587 | Completed | Icahn school of medicine at Mount Sinai |
| Pexa-Vec<br>Ipilimumab | I | Metastatic tumor<br>Advanced tumor | NCT02977156 | Completed | Centre Leon Berard |
| GL-ONC1<br>Radiation<br>Cisplatin | I | Head and neck cancer | NCT01584284 | Completed | Moores UC San Diego cancer center |
| REOLYSIN®<br>Irinotecan<br>Leucovorin<br>Fluorouracil (5-FU)<br>Bevacizumab | I | KRAS mutant metastatic colorectal cancer | NCT01274624 | Completed | Oncolytics biotech and montefiore medical center |
| REOLYSIN®<br>Gemcitabine<br>Irinotecan<br>Leucovorin<br>5-fluorouracil<br>Pembrolizumab | I | Pancreatic adenocarcinoma | NCT02620423 | Completed | Cancer therapy and research center at UTHSCSA |
| GL-ONC1<br>Bevacizumab | I/II | Ovarian cancer<br>Peritoneal carcinomatosis<br>Fallopian tube cancer | NCT02759588 | Completed | Genelux corporation |
| Ad-MAGEA3<br>MG1-MAGEA3<br>Pembrolizumab | I/II | Non-small cell lung cancer | NCT02879760 | Completed | Turnstone biologics, Corp. |
| TBI-1401(HF10)<br>Ipilimumab | II | Melanoma stage III<br>Melanoma stage IV | NCT03153085 | Completed | National cancer center hospital |
| DNX-2401<br>Pembrolizumab | II | Brain cancer<br>Brain neoplasm<br>Glioma<br>Glioblastoma<br>Gliosarcoma<br>Malignant brain tumor<br>Neoplasm,<br>Neuroepithelial<br>neuroectodermal tumors | NCT02798406 | Completed | DNAtrix, Inc. |

| | | | | | |
|---|---|---|---|---|---|
| | | Neoplasm by histologic type<br>Nerve tissue nervous system diseases | | | |
| HF10<br>Ipilimumab | II | Malignant melanoma | NCT02272855 | Completed | University of Utah |
| RT-01<br>Nivolumab | I | Advanced solid tumor | NCT05228119 | Recruiting | The affiliated hospital of Xuzhou medical university |
| RT-01<br>Nivolumab | I | Advanced solid tumor | NCT05122572 | Recruiting | Wuxi People's hospital |
| ASP9801<br>Pembrolizumab | I | Metastatic cancer<br>Solid tumor<br>Advanced cancer | NCT03954067 | Recruiting | Astellas pharma global development, Inc. |
| RP3<br>Nivolumab | I | Advanced solid tumor | NCT04735978 | Recruiting | Replimune Inc. |
| Enadenotucirev<br>Capecitabine<br>Radiotherapy | I | Locally advanced rectal cancer | NCT03916510 | Recruiting | University of Oxford |
| TILT-123<br>Avelumab | I | Melanoma<br>Head and neck squamous cell Carcinoma | NCT05222932 | Recruiting | Docrates cancer center |
| OVV-01 injection<br>Pembrolizumab | I | Neoplasms | NCT04787003 | Recruiting | North China petroleum bureau general hospital |
| TBio-6517<br>Pembrolizumab | I/II | Solid tumor<br>Microsatellite stable colorectal cancer<br>HPV-positive oropharyngeal squamous cell carcinoma<br>Cervical cancer<br>Melanoma (skin)<br>Cutaneous squamous cell carcinoma<br>Mesothelioma<br>Renal cell carcinoma<br>Oropharynx cancer | NCT04301011 | Recruiting | Turnstone biologics |
| Delolimogene<br>Mupadenorepvec<br>Gemcitabine | I/II | Pancreatic cancer | NCT02705196 | Recruiting | Lokon pharma AB |

| | | | | | |
|---|---|---|---|---|---|
| Nab-Paclitaxel<br>Atezolizumab | | | | | |
| Olvimulogene Nanivacirepvec<br>Pcarboplatin (preferred) or Cisplatin<br>Gemcitabine,<br>Taxane (Paclitaxel, Docetaxel or Nab-Paclitaxel) or<br>Pegylated liposomal Doxorubicin<br>Bevacizumab | III | Platinum-resistant ovarian cancer<br>Platinum-refractory ovarian cancer<br>Fallopian tube cancer<br>Primary peritoneal cancer<br>High-grade serous ovarian cancer<br>Endometrioid ovarian cancer<br>Ovarian clear cell carcinoma | NCT05281471 | Recruiting | Genelux corporation<br>GOG foundation |
| MEDI5395<br>Durvalumab | I | Advanced solid tumors | NCT03889275 | Active not recruiting | MedImmune LLC |
| ONCR-177<br>Pembrolizumab | I | Melanoma<br>Solid tumor<br>Squamous cell carcinoma of head and neck<br>Breast cancer<br>Advanced solid tumor<br>Triple negative breast cancer<br>Colorectal carcinoma<br>Non-melanoma skin cancer<br>Liver metastases | NCT04348916 | Active not recruiting | Oncorus, Inc. |
| ADV/HSV-tk<br>Valacyclovir<br>Stereotactic body radiation therapy<br>Pembrolizumab | I | Metastatic non-small cell lung cancer<br>Metastatic triple-negative breast cancer | NCT03004183 | Active not recruiting | Houston methodist cancer center |
| TBI-1401(HF10)<br>Gemcitabine<br>Nab-paclitaxel<br>TS-1 | I | Pancreatic cancer stage III<br>Pancreatic cancer stage IV | NCT03252808 | Active not recruiting | Takara Bio Inc. |
| Pexastimogene Devacirepvec (Pexa-Vec)<br>Cemiplimab | I/II | Renal cell carcinoma | NCT03294083 | Active not recruiting | SillaJen, Inc.<br>Regeneron pharmaceuticals |
| Talimogene laherparepvec<br>Paclitaxel | I/II | Ductal carcinoma<br>Invasive breast carcinoma<br>Invasive ductal breast carcinoma | NCT02779855 | Active not recruiting | H. Lee Moffitt cancer center and research institute |

| | | | | | |
|---|---|---|---|---|---|
| Durvalumab<br>Tremelimumab<br>Pexa-Vec | I/II | Colorectal cancer<br>Colorectal carcinoma<br>Colorectal adenocarcinoma<br>Refractory cancer<br>Colorectal neoplasms | NCT03206073 | Active not recruiting | National cancer institute (NCI) |
| Paclitaxel<br>Pelareorep<br>Avelumab | II | Breast cancer metastatic | NCT04215146 | Active not recruiting | Oncolytics biotech<br>PrECOG, LLC. |
| Recombinant oncoly tic HSV-2<br>Recombinant humanized anti-PD-1 mAb<br>Radiation | I | Melanoma stage IV | NCT05068453 | Not yet recruiting | Peking university cancer hospital & institute |
| Recombinant oncoly tic HSV-2<br>Axitinib | I | Melanoma stage IV | NCT05070221 | Not yet recruiting | Peking university cancer hospital & institute |
| Revottack<br>PD-1 | I | Advanced solid tumor | NCT05644509 | Not yet recruiting | The affiliated hospital of Xuzhou medical university |
| G207<br>Radiation (5 Gy single dose) | II | High-grade glioma<br>Glioblastoma multiforme<br>Malignant glioma of the brain<br>Anaplastic astrocytoma of the brain<br>High-grade glioma<br>Anaplastic glioma<br>Giant cell glioblastoma | NCT04482933 | Not yet recruiting | University of Alabama at Birmingham |
| Lenalidomide or Pomalidomide<br>REOLYSIN | I | Multiple myeloma | NCT03015922 | Unknown | St. James's university hospital |
| TG6002<br>5-flucytosine | I/II | Glioblastoma<br>Brain cancer | NCT03294486 | Unknown | Assistance Publique - Hôpitaux de Paris |
| H101<br>Radiation | - | Genital neoplasms | NCT05051696 | Enrolling by invitation | First affiliated hospital Xi'an Jiaotong university |

**Supplementary Table S2. Recent FDA-approved small molecules targeting essential pathways for cancer treatment**

| Drug name | Active small molecule | Structure | Target | Cancer type | Approval date |
|---|---|---|---|---|---|
| Balversa | Erdafitinib | 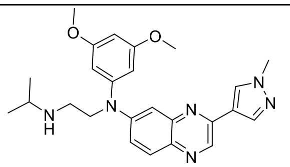 | pan-EGFR inhibitor | Advanced and metastatic bladder cancer | 4/12/2019 |
| Piqray | Alpelisib | 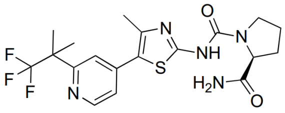 | PI3K-$\alpha$ inhibitor | Breast cancer | 5/24/2019 |
| Xpovio | Selinexor | 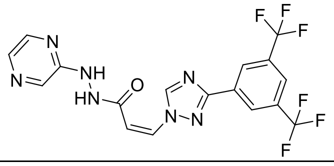 | XPO1 inhibitor | Relapsed and refractory myeloma | 7/3/2019 |
| Nubeqa | Darolutamide | 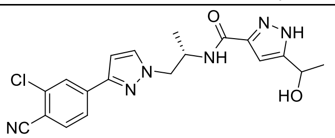 | AR antagonist | Non-metastatic castration resistant prostate cancer | 07/30/2019 |
| Turalio | Pexidartinib | 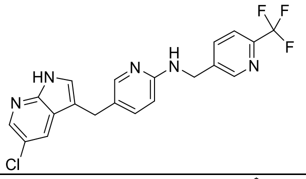 | CSFIR inhibitor | Symptomatic tenosynovial giant cell tumor | 08/02/2019 |
| Rozlytrek | Entrectinib | 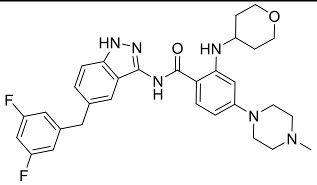 | ROS1/TRK/AL K inhibitor | Metastatic non-small cell lung cancer | 08/15/2019 |
| Inrebic | Fedratinib | 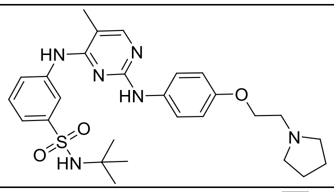 | JAK-2 inhibitor | High-risk primary and secondary myelofibrosis | 08/16/2019 |
| Brukinsa | Zanubrutinib | 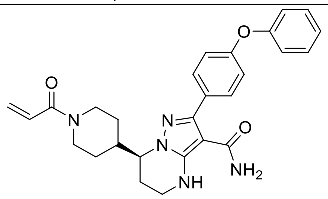 | BTK inhibitor | Mantle cell lymphoma | 11/14/2019 |
| Tazverik | Tazemetostat | 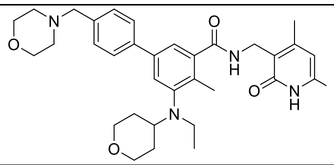 | EZH2 methyl transferase inhibitor | Epitheloid carcinoma | 1/23/2020 |

| | | | | | |
|---|---|---|---|---|---|
| Koselgo | Selumetinib | 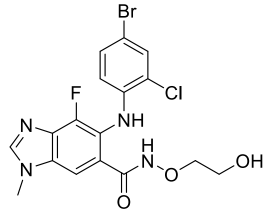 | MEK1/MEK2 kinase inhibitor | Neurofibromatosis type 1 | 4/10/2020 |
| Retevmo | Selpercatinib | 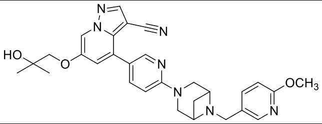 | RET fusion kinase Inhibitor | NSCLC, Thyroid cancer | 5/8/2020 |
| Zepzelca | Iurbinectedin | 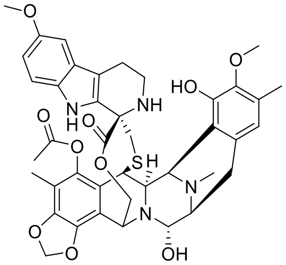 | DNA alkylating agent | Metastatic small cell lung cancer | 6/15/2020 |
| Inqovil | Cedazuridine/ Decitabine | 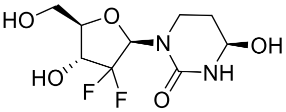 | Nucleoside cytidine deaminase inhibitor | Myelodysplastic syndromes | 7/7/2020 |
| Orgovyx | Relugolix | 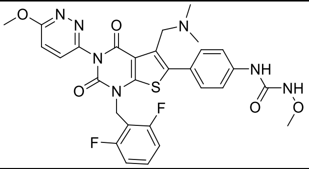 | GnRH hormone receptor antagonist | Advanced prostate cancer | 12/18/2020 |
| Tepmetko | Tepotinib | 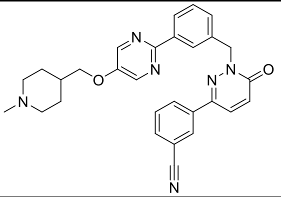 | MET kinase inhibitor | NSCLC | 2/3/2021 |
| Ukoniq | Umbralisib | 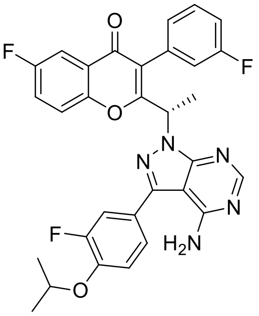 | PI3Kδ and CK1€ Kinase inhibitor | Follicular lymphoma | 2/5/2021 |
| Cosela | Trilaciclib | 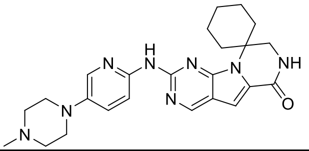 | CDK-4 and CDK-5 Kinase inhibitor | SCLC | 2/12/2021 |

| | | | | | |
|---|---|---|---|---|---|
| Pepaxto | Melphalan-flufenamide | 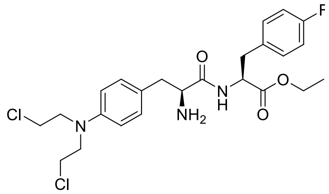 | Conjugated alkylating agent | Multiple myeloma | 2/26/2021 |
| Fotivda | Tivozanib | 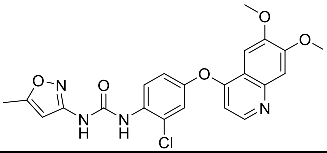 | Pan-VEGFR kinase inhibitor | Renal cell carcinoma | 3/10/2021 |
| Lumakras | Sotorasib | 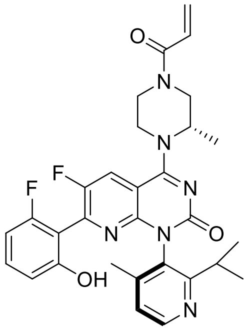 | KRAS GTPase inhibitor | NSCLC | 5/21/2021 |
| Truseltiq | Infigratinib | 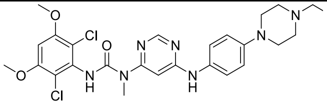 | Pan-FGFR kinase inhibitor | Cholangiocarcinoma | 5/28/2021 |
| Exkivity | Mobocertinib | 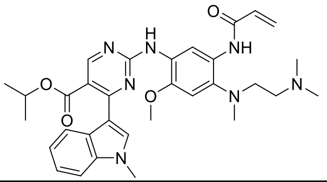 | EGF Rexon 20 insertion kinase inhibitor | NSCLC | 9/15/2021 |
| Scemblix | Asciminib | 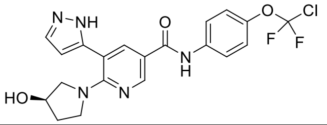 | ABL/BCR-ABL1 kinase inhibitor | Myeloid leukemia | 10/29/2021 |
| Pluvicto | Lutetium (177Lu) vipivotide tetraxetan | 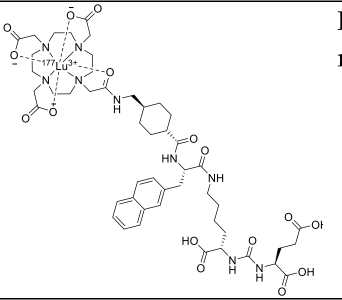 | PSMA targeted radiotherapy | Prostate cancer | 3/23/2022 |
| Vonjo | Pacritinib | 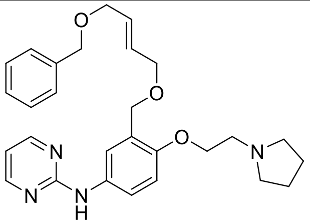 | JAK-2/IRAK1 inhibitor | Myelofibrosis | 2/28/2022 |

| | | | | | |
|---|---|---|---|---|---|
| Lytgobi | Futibatinib | 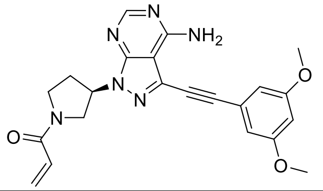 | FGFR2 inhibitor | Intrahepatic cholangiocarcinoma | 9/30/2022 |
| Rezlidhia | Olutasidenib | 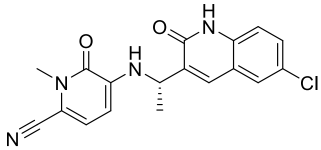 | IDG1 inhibitor | Relapsed/ refractory acute myeloid leukemia | 12/1/2022 |
| Krazati | Adagrasib | 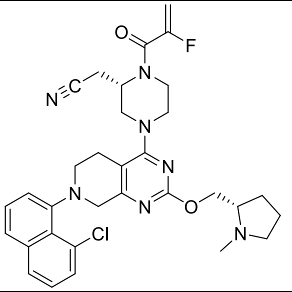 | KRAS inhibitor | Advanced or metastatic NSCLC | 12/12/2022 |
